# Supplementary material for: The Impact of Minimal Intervention Dentistry on Patient-Reported and Observation-Based Outcomes in the Pediatric Population: A Systematic Review and Meta-Analysis
Source: Healthcare (Basel). 2023 Aug 9;11(16):2241. doi: 10.3390/healthcare11162241 (PMC10454713; doi:10.3390/healthcare11162241)
Supplement: Supplementary file 1 [file healthcare-11-02241-s001.zip › healthcare-2522936-supplementary.pdf]

The impact of Minimal Intervention Dentistry on patient-reported and observation-based outcomes in the paediatric population: a systematic review and meta-analysis

Key words: minimal intervention dentistry, patient-reported outcomes, dental fear and anxiety, children, systematic review, meta-analysis

Supplementary material

Figure S1. Meta-analysis. HT vs CSSC. Pain and anxiety level.

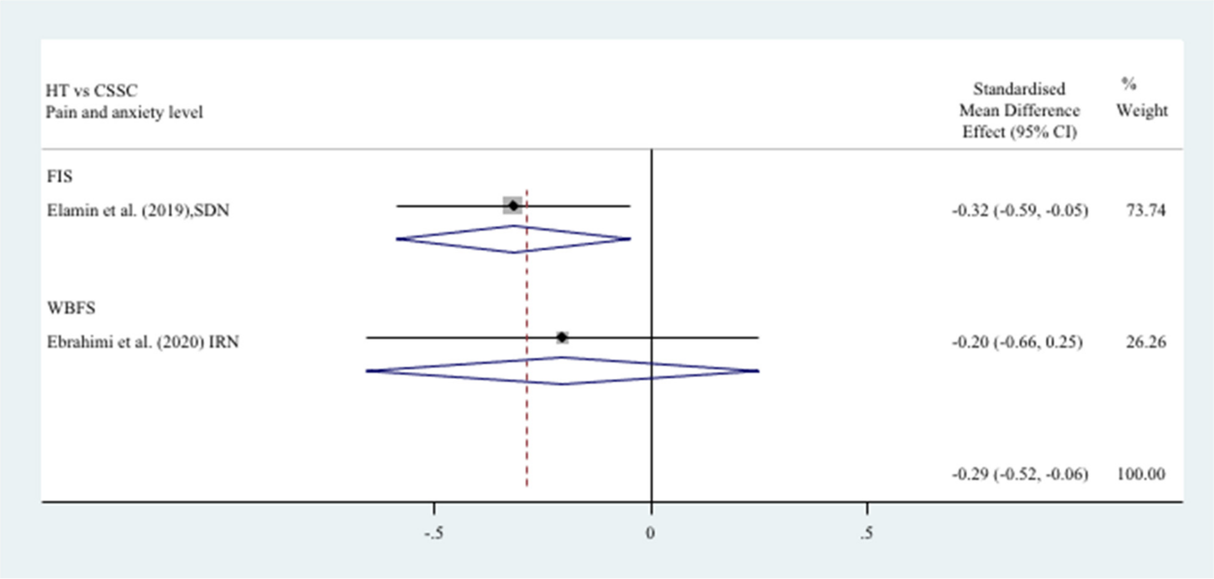

Figure S2. Meta-analysis. HT vs CSSC. time

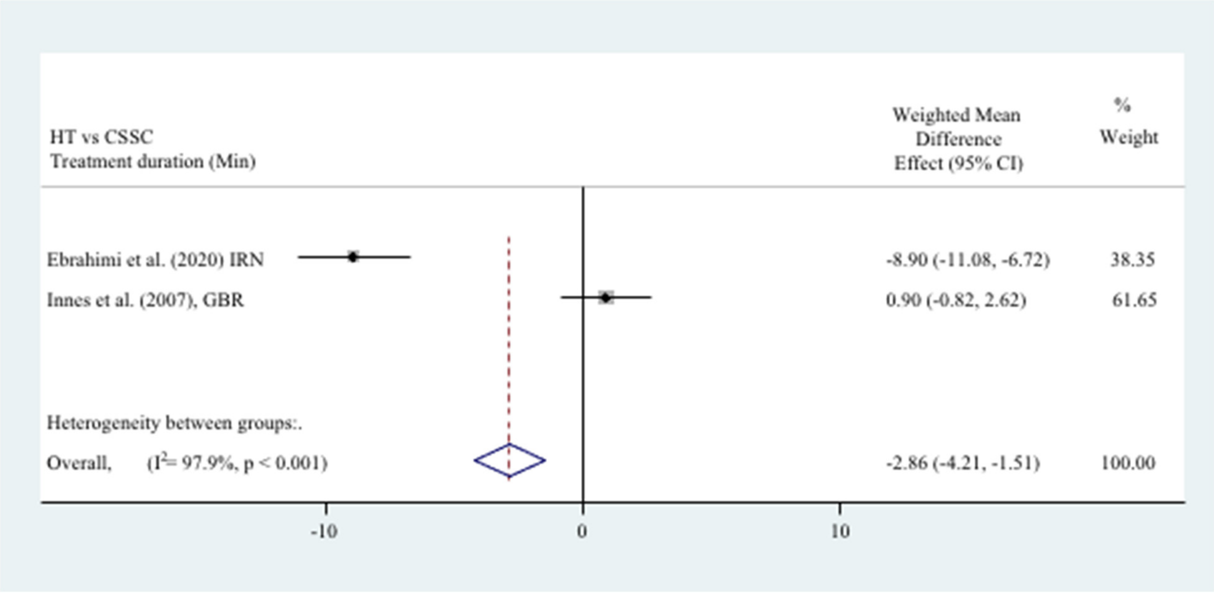

Table S1. Search Strategy

[dental] AND [anxiety] OR [fear] OR [phobia] OR [apprehension] AND [paediatric] OR [child] OR [children] OR [preschool] AND [caries] OR [carious removal] AND [non-invasive] OR [fluoride] OR [sealant] OR [resin infiltration] OR [minimally invasive] OR [ART] OR [atraumatic restorative technique] OR [IRT] OR [interim restorative technique] OR [caries stabilization] OR [partial caries removal] OR [indirect pulp treatment] OR [hand excavation] AND [randomized control trial] OR [RCT] OR [controlled clinical trial] OR [CCT] or [quasi trial].

Table S2. Excluded studies after full text screening

No.	Reasons for exclusion	Studies
1	No English full text	Actrn, Effectiveness of treating of early childhood dental decay using minimally invasive Atraumatic Restorative Treatment-based approach. http://www.who.int/trialsearch/Trial2.aspx?TrialID=ACTRN12616001124426, 2016.
2	Reporting oral health related quality of life	Arrow, P. and H. Forrest, Atraumatic restorative treatments improve child oral health-related quality of life: A noninferiority randomized controlled trial. Community dentistry and oral epidemiology, 2020. 48(4): p. 349-356.
3	Reporting oral health related quality of life	Arrow, P. and E. Klobas, Minimum intervention dentistry approach to managing early childhood caries: a randomized control trial. Community dentistry and oral epidemiology, 2015. 43(6): p. 511-520.
4	Reporting oral health related quality of life	Arrow, P. and E. Klobas, Child oral health-related quality of life and early childhood caries: a non-inferiority randomized control trial. Australian Dental Journal, 2016. 61(2): p. 227-235.
5	Registered protocol	Arrow, P., et al., Minimally Invasive Dentistry Based on Atraumatic Restorative Treatment to Manage Early Childhood Caries in Rural and Remote Aboriginal Communities: Protocol for a Randomized Controlled Trial. JMIR Res Protoc, 2018. 7(7): p. e10322.
6	Outcome not patient/observer based	Arrow, P., et al., Atraumatic restorative treatments and oral health-related quality of life and dental anxiety in Australian Aboriginal children: A cluster-randomized trial. Community Dentistry and Oral Epidemiology: p. 9.
7	No English full text	Dmitrova, A.G. and A.A. Kulakov, The atraumatic restorative treatment approach in pediatric dental care: a comparative clinical study. [Russian]. Stomatologiia, 2015. 94(2): p. 30-33.
8	Irrelevance	Duangthip, D., Fung, M. H. T., Wong, M. C. M., Chu, C. H., & Lo, E. C. M. (2018). Adverse Effects of Silver Diamine Fluoride

		Treatment among Preschool Children. Journal of dental research, 97(4), 395–401. https://doi.org/10.1177/0022034517746678
9	No English full text	Estupiñan-Day 2006 {published data only} * Estupiñan-Day S, Milner T, Tellez M. Oral health of low income children: procedures for atraumatic restorative treatment (PRAT). Pan American Health Organization 2006. Estupiñán-Day S, Tellez M, Kaur S, Milner T, Solari A. Managing dental caries with atraumatic restorative treatment in children: successful experience in three Latin American countries. Revista Panamericana de Salud Publica 2013;33(4):237-43
10	Non-randomised controlled trial/ observational study	Jackson G. PLACEMENT OF PREFORMED METAL CROWNS ON CARIOUS PRIMARY MOLARS BY DENTAL HYGIENE/THERAPY VOCATIONAL TRAINEES IN SCOTLAND: A SERVICE EVALUATION ASSESSING PATIENT AND PARENT SATISFACTION. Prim Dent J. 2015 Nov;4(4):46-51. doi: 10.1308/205016815816682218. PMID: 26966773
11	Reporting caries prevention and clinical success	Jiang, M., et al., Effects of restoring SDF-treated and untreated dentine caries lesions on parental satisfaction and oral health related quality of life of preschool children. Journal of dentistry, 2019. 88: p. 103171.
12	Reporting caries prevention and clinical success	Jiang, M., et al., Association between dental conditions, sliver diamine fluoride application, parental satisfaction, and oral health-related quality of life of preschool children. Clinical oral investigations, 2021. 25(2): p. 653-662.
13	Reporting oral health related quality of life	Leal, S.C., et al., Effect of different protocols for treating cavities in primary molars on the quality of life of children in Brazil - 1 year follow-up. International Dental Journal, 2013. 63(6): p. 329-335.
14	No English full text	Ling 2003 {published data only} Ling L, Wang X. Evaluation of effects of Atraumatic Restorative Treatment and cooperation degree in primary teeth. Stomatology 2003;23(5):290-91.
15	Subjects over 12 years old	Lopez N, Simpser-Rafalin S, Berthold P. Atraumatic restorative treatment for prevention and treatment of caries in an underserved community. Am J Public Health. 2005 Aug;95(8):1338-9. doi: 10.2105/AJPH.2004.056945. Epub 2005 Jul 7. PMID: 16006415; PMCID: PMC1449363. (participants: 18 years)
16	Non-randomised controlled trial/ observational study	Louw AJ, Sarvan I, Chikte UM, Honkala E. One-year evaluation of atraumatic restorative treatment and minimum intervention techniques on primary teeth. SADJ. 2002 Sep;57(9):366-71. PMID: 12494713.
17	Reporting caries prevention and clinical success	Maguire, A., et al., Best-practice prevention alone or with conventional or biological caries management for 3- to 7-year-olds: The fiction three-arm RCT. Health Technology Assessment, 2020. 24(1): p. vii-174.

18	Outcome not patient/observer based	Maciel R, Salvador D, Azoubel K, Redivivo R, Maciel C, da Franca C, Amerongen E, Colares V. The opinion of children and their parents about four different types of dental restorations in a public health service in Brazil. Eur Arch Paediatr Dent. 2017 Feb;18(1):25-29. doi: 10.1007/s40368-016-0262-8. Epub 2017 Jan 2. PMID: 28044248. (the children did not receive the restorations)
19	Reporting oral health related quality of life	Mashoto, K.O., et al., Changes in the quality of life of Tanzanian school children after treatment interventions using the Child-OIDP. European Journal of Oral Sciences, 2010. 118(6): p. 626-634.
20	Reporting caries prevention and clinical success	Maguire, A., Clarkson, J. E., Douglas, G. V., Ryan, V., Homer, T., Marshman, Z., McColl, E., Wilson, N., Vale, L., Robertson, M., Abouhajar, A., Holmes, R. D., Freeman, R., Chadwick, B., Deery, C., Wong, F., & Innes, N. P. (2020). Best-practice prevention alone or with conventional or biological caries management for 3- to 7-year-olds: the FiCTION three-arm RCT. Health technology assessment (Winchester, England), 24(1), 1–174. https://doi.org/10.3310/hta24010
21	Outcome not patient/observer based	Mickenautsch, S., J.E. Frencken, and H.M. van't, Atraumatic restorative treatment and dental anxiety in outpatients attending public oral health clinics in South Africa. J Public Health Dent, 2007. 67(3): p. 179-84.
22	Reporting oral health related quality of life	Mijan, M.C., et al., Children's Oral Health-related Quality of Life (OHRQoL) Three Years after Implementation of Treatment Protocols for Managing Cavitated Carious Dentine Lesions. Oral Health Prev Dent, 2019. 17(1): p. 83-89.
23	Reporting caries prevention and clinical success	Rodrigues, G.F., et al., Oral Health-Related Quality of Life in Preschool Children After Silver Diamine Fluoride Versus Atraumatic Restorative Treatments. Pediatr Dent, 2020. 42(5): p. 373-379.
24	Special Healthcare needs patients	15. Robertson MD, Harris JC, Radford JR, Innes NPT. Clinical and patient-reported outcomes in children with learning disabilities treated using the Hall Technique: a cohort study. Br Dent J. 2020 Jan;228(2):93-97. doi: 10.1038/s41415-019-1166-x. PMID: 31980784.
25	Irrelevance	Roshan, N.M. and B. Sakeenabi, Anxiety in children during occlusal ART restorations in primary molars placed in school environment and hospital dental setup. J Clin Pediatr Dent, 2012. 36(4): p. 349-52.
26	Reporting oral health related quality of life	Ruff, R.R., et al., Silver diamine fluoride, atraumatic restorations, and oral health-related quality of life in children aged 5-13 years: results from the CariedAway school-based cluster randomized trial. BMC oral health, 2022. 22(1): p. 125.

27	Reporting oral health related quality of life	Sihra, R., et al., The Effectiveness of Silver Diamine Fluoride and Fluoride Varnish in Arresting Caries in Young Children and Associated Oral Health-Related Quality of Life. Journal (Canadian Dental Association), 2020. 86: p. k9.
28	No English full text	Wang, J. and H. Wang, Study of the consistency for three means to evaluate children's dental fear. [Chinese]. Shanghai kou qiang yi xue = Shanghai journal of stomatology, 2006. 15(6): p. 581-584.
29	No English full text	Wang, J. and H. Wang, Effects of three caries removal methods on children's dental fear evaluated by physiological measure. [Chinese]. Shanghai kou qiang yi xue = Shanghai journal of stomatology, 2007. 16(2): p. 149-152.
30	No English full text	Wang, J. and H.M. Wang, [Effects of three caries removal methods on children's dental fear evaluated by physiological measure]. Shanghai Kou Qiang Yi Xue, 2007. 16(2): p. 149-52.

Table S3. Summary of findings of comparisons with ART

	Study (Year & Country)	N Participants (% M); range range (mean)	RCT design, settings	Method of assessment	Intervention group/ control groups	Results
ART vs CR						
1	Arrow; E. Klobas (2017) AUS	254 (59%) ; 3.7-3.9 (3.8)	Parallel group, dental clinic	(1)Facial Image scale	(1) ART<br><br>(2) Conventional treatment with LA and RD	Similar anxiety
2	Abreu et al. (2011) BRA	302 (55%); 6-7 years old (6.8)	3 parallel groups, dental clinic	(1)Facial Image scale	(1) ART<br><br>(2) Conventional restorative treatment (LA +rotary instruments)	Similar anxiety
3	Abreu et al. (2011) BRA	244 (57%) 6- 7 years old (6.8)	3 parallel groups, dental clinic	(1) Wong Baker FACES pain rating scale	(1) ART<br><br>(2) Conventional restorative treatment with LA without RD	Similar pain, but more children needs LA
4	Eden et al. (2006) NLD	157 (48%) 7 years old (7.0)	Split mouth design with washout, University dental clinic	(1) Time taken	(1) ART<br><br>(2) Convential restoration without LA	Time shorter for CR

5	Luz et al. (2012) BRA	30 (43.3), 4-7 years old (NR)	2 parallel groups, clinic study	(1) Time taken	(1) ART<br><br>(2) Conventional restorative treatment with LA without RD	Time longer for CR
6	Shricks et al. (2003) IDN	403 (51.6%); 4-7 years old (6.3)	2 parallel groups, hospital dental clinic	(1) Venham index	(1) ART restoration<br><br>(2) Conventional restorations without LA without RD	Significantly lower anxiety with ART group
7	Tavares et al. (2017) BRA	79 (36.7%) ; 5-8 years old (6.6)	Split mouth design with washout, University dental clinic	(1) Facial Image scale 1-5 scale<br>(2) Wong Baker Faces scale	(1) Conventional restorations without LA without RD<br><br>(2) ART restoration	Significantly less discomfort with ART
8	Topaloglu-Ak et al. 2007 TUR	160 (N/A) 6-7 years old	2 parallel groups, university dental clinic	1(venham picture test)	(1) Conventional restorations without LA without RD<br>(2) ART restorations	No difference in time and anxiety
9	Van Bochove et al. (2006)NLD	300 (48%) ; 6-7 years old (6.98)	4 parallel groups, university dental clinic	(1) Venham index<br>(2) Venham Picture Test	(1) Conventional restorations with LA<br><br>(2) Conventional restorations without LA<br>(3) ART with LA	Least discomfort with ART without LA; most discomfort with CR with LA

					(4) ART without LA	
10	van de Hoef (2007), NLD	299 (51.8) 6-7 years (7.5)	4 parallel groups, university dental clinic	(1) Venham index	(1) Conventional restorations with LA<br>(2) Conventional restorations without LA<br>(3) ART with LA<br>(4) ART without LA	No difference in anxiety between the 4 groups
ART vs cavity modification						
1	Abreu et al. (2011) Brazil	302 (55%); 6-7 years old (6.8)	3 parallel groups, dental clinic	(1)Facial Image scale	(1) ART<br>(2) Cavity modifications	No difference
2	Abreu et al. (2011) Brazil	244 (57%) 6- 7 years old (6.8)	3 parallel groups, dental clinic	(1) Wong Baker FACES pain rating scale	(1) ART<br>(2) Cavity modifications	No difference
3	Goud et al. (2012) India	200 (no data); 6-8 years old (no mean provided)	Parallel group, Hospital dental clinic	(1) Venham Scale	(1) ART restoration<br>(2) Non-invasive treatment (cavity modification)	Less discomfort with ART than cavity modification

Table S4. Summary of findings of comparisons with HT

No.	Study (Year & Country)	N Participants (% M); range range (mean)	RCT design, settings	Method of assessment	Intervention group/ control groups	Results
	HT vs CSSC anxiety scale					
1	Elamin et al. (2019) Sudan	164 (50.4), 5-8 years old (NR)	Parallel groups, General dental clinic	(1) Facial Image Scale	(1) Hall's Technique (HT)<br><br>(2) Conventional SSC placement (CT)	HT less anxiety than CT
2	Ebrahimi et al. (2020) Iran	123 (37.4), 4-9 years old	3 parallel groups, university dental clinic	(1) FACES Pain Scale-revised	(1) Hall's Technique (HT)<br>(2) Conventional SSC placement (CT)<br>(3) GIS restoration	No difference in child anxiety<br>More cooperative for CSSC
	HT vs conventional SSC, time					
1	Ebrahimi et al. (2020) Iran	123 (37.4), 4-9 years old	3 parallel groups, university dental clinic	Time taken	(1) Hall's Technique (HT)<br>(2) Conventional SSC placement (CT)<br>(3) GIS restoration	Longer for SSC
2	Innes et al. (2007), UK	132 (52.2) 3-10 years (6.8)	split mouth, general dental practice	Time taken	(1) Hall' Technique<br>(2) Conventional SSC	No difference in time
	HT vs CR, cooperativeness					

1	Santamaria et al. (2014) Germany	169 (56.8%); 3-8 years old (5.55)	3 parallel groups, university dental clinic	(1) Frankl Scale (2) Visual Analogue scale of faces (5 point scale)	(1) Conventional restorations (2) Hall's Technique SSC (3) Non-invasive treatment (cavity modification)	No difference in pain More uncooperative for CR
2	Thakkar &Jawdekar (2022) India	60 (48%), 7-8 years old (7.6)	2 parallel groups, university dental clinic	(1) Frankl Scale (2) Time	(1) HT (2) Conventional restorations	No difference in cooperativeness
	HT vs ART, pain and anxiety					
1	Araujo et al. (2020) Brazil	131 (N/A); 5-10 years old (8.1)	2 parallel groups (randomized), university dental clinic	(1) Wong Baker FACES pain rating scale	(1) ART restoration (2) Hall's Technique SSC placement	More discomfort for HT
2	Ebrahimi et al. (2020) Iran	123 (37.4), 4-9 years old	3 parallel groups, university dental clinic	(1) FACES Pain Scale-revised (2) Time	(1) Hall's Technique (HT) (2) Conventional SSC placement (CT) (3) GIS restoration	No difference in anxiety
3	Lakshmi et al. (2018) India	30 (NR); 5-8 years old (NR)	2 parallel groups, clinic study	(1) Wong Baker Faces Scale	(1) ART restoration (2) Hall technique SSC	No difference in pain
